# Sex, male origin microchimerism, and mortality in a Danish cohort

**DOI:** 10.1007/s10654-025-01309-7

**Published:** 2025-09-24

**Authors:** Gitte Lindved Petersen, Tri-Long Nguyen, Rune Lindahl-Jacobsen, Anne Tjønneland, Mads Kamper-Jørgensen

**Affiliations:** 1 Department of Translational Type 1 Diabetes Research, Steno Diabetes Center Copenhagen, Herlev, Denmark; 2 Section of Epidemiology, Department of Public Health, University of Copenhagen, Copenhagen, Denmark; 3 Unit of Epidemiology, Biostatistics and Biodemography, Department of Public Health, University of Southern Denmark, Odense, Denmark; 4 Interdisciplinary Centre on Population Dynamics, University of Southern Denmark, Odense, Denmark; 5 Danish Cancer Institute, Copenhagen, Denmark; 6 Section of Environmental Health, Department of Public Health, University of Copenhagen, Copenhagen, Denmark

**Keywords:** Male origin microchimerism, Mortality, Prognostic score weighing, Restricted mean survival time difference, Causal inference, Denmark

## Abstract

We previously demonstrated that women with detectable male cells in peripheral blood – male origin microchimerism (a presumed souvenir from pregnancy) – enjoy half the risk of all-cause mortality compared with women tested negative. However, such differences are vulnerable to confounding. Also, it is unknown whether better survival in women vs. men is confined to women testing male origin microchimerism positive. In this updated analysis, we first compared the survival of positive and negative women using prognostic score weighing to minimize potential confounding. Next, we compared the survival of women tested positive and negative for male origin microchimerism to men. Women (*n* = 766) and men (*n* = 1,000) from the Diet, Cancer, and Health cohort aged 50–64 years in 1993–1997 were followed until 2017 for all-cause mortality in national Danish registers. Based on predictors of death, we calculated prognostic scores for all women and compared mortality according to male origin microchimerism status by prognostic score weighted Cox regression adjusting for potential confounding. Next, we compared mortality across positive/negative women and men. Women who tested male origin microchimerism positive versus negative had a 50% lower mortality rate (adjusted HR = 0.50 [95% CI 0.32–0.77]). Compared with men, women who tested positive had a 51% lower mortality rate (HR = 0.49 [95% confidence interval (CI) 0.38–0.61]), whereas women who tested negative had a similar rate (HR = 0.83 [95% CI 0.63–1.09]). Women who tested positive for male origin microchimerism lived longer than their counterparts who tested negative, who appeared to have a similar survival pattern to that of men.

## Introduction

In most societies women outlive men [1], and parenthood is associated with reduced mortality in men as well as women [2]. In men, however, this survival benefit attenuates after adjusting for health behaviors, whereas this is not the case for women [3]. Based on the notion that pregnancy and childbirth are positively associated with longevity [4–6], we previously demonstrated that women with persistent presence of male cells in peripheral blood, likely obtained during pregnancy – so-called male origin microchimerism (MOM) [7] – enjoy half the risk of all-cause mortality compared with women without MOM [8]. MOM is thought to be a souvenir from pregnancy with a male fetus [7]. However, its detection in 24% of women without sons [9] and 14% of young girls [10], suggest alternative sources. One hypothesis posits that male cells originate back to maternal circulation, potentially transferred across generations [11]. Other proposed sources include previous abortions, vanished male twin gestations, or sexual intercourse [9]. Despite the marked difference demonstrated in our previous study, the sample was limited to 272 women with 22 deaths and no men, which did not facilitate analyses of the total effect of sex, nor firm conclusions about the potential causal effect of MOM on survival in women, especially not for subgroups. Finally, unknown confounding could have biased the association estimates.

Here, we present an updated analysis of the role of MOM on mortality in an additional 502 women, according to presence or absence of MOM, and in 1,000 men from the same cohort, followed up for mortality for a further eight years. Our updated comparison between MOM positive and negative women addresses the risk of confounding by weighing for prognostic scores. Also, for the first time, we compare the mortality of MOM positive women, MOM negative women, and men.

## Material and methods

### Material

Our original study of the association between MOM and mortality in women was carried out as a cohort study using peripheral blood samples and questionnaire data (including lifestyle, height, weight, and parity) from 272 women randomly sampled from the Danish Diet, Cancer and Health (DCH) cohort [12], linked with nationwide registry data (including date and cause of death). Women were followed up for all-cause mortality until the end of 2009. To study associations between MOM and female malignancies [13–15] and cardiovascular [16] outcomes, we subsequently obtained peripheral blood samples and questionnaire data for an additional 494 randomly sampled women from the DCH cohort. A total of 774 women had available MOM exposure data from laboratory analysis of blood samples, confounding variable data from DCH questionnaires, and all-cause mortality data from the Danish Civil Registration System [17] until September 19, 2017. Only women with complete information on the baseline variables used for constructing the prognostic scores were included in the study (*n* = 766). In addition, we included a random sample of 1,000 men participating in the DCH cohort. For men, we had no measure of MOM, so we only included variables from the DCH baseline questionnaire for descriptive comparison with women and register-based mortality until September 19, 2017.

### Laboratory methods

We obtained 1.2 *μg* DNA purified from buffy coat equivalent to 180,000 maternal cells from each woman. To identify presence of male DNA, we applied a validated qPCR assay targeting Y-chromosome gene *DYS14* sequences [18], which should never be present in women. Samples were run in 6-plicate with DNA corresponding to 30,000 cells per well. qPCR was performed as previously described by our group [10]. We set the threshold cycle to 40, and if the intensity of the fluorescent light from a minimum of one of six wells crossed the threshold, the test was considered positive for the presence of MOM. Six wells with no template controls (NTC) were included for each run, and for a run to be accepted, all six NTC should be negative. We dichotomized the presence of MOM as positive versus negative by denoting a sample positive if at least one well tested positive, and negative if all wells proved negative. The estimated concentration of MOM was categorized as 0, > 0 to < 2, ≥2 to < 4, and ≥ 4 MOM cells per 10⁶ cells tested. To avoid male contamination, laboratory analyses were performed by female technicians who were blinded to the case/control status of the woman from whom the sample was taken.

### Statistical methods

**Fig. 1:**
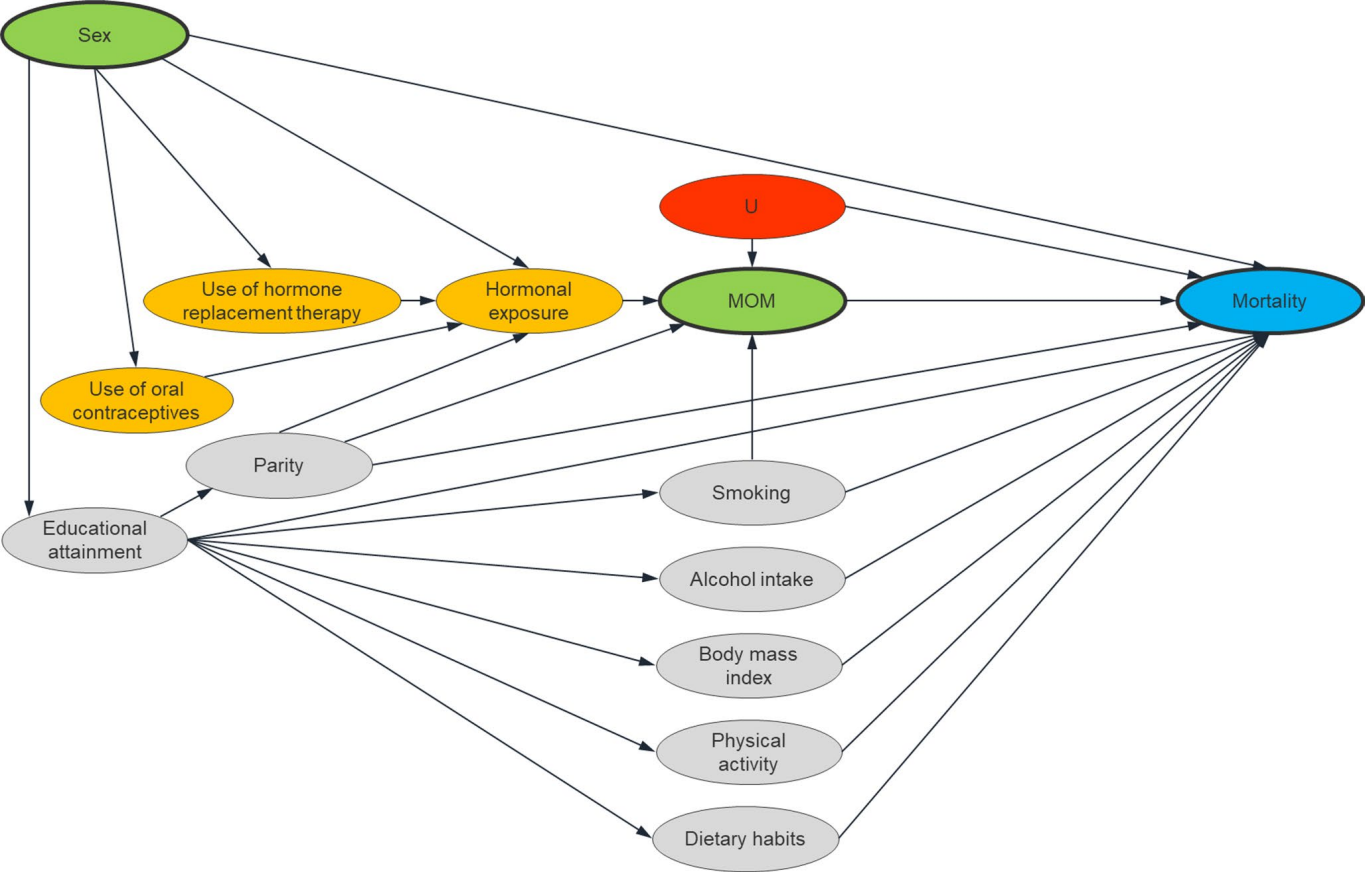
Directed Acyclic graph depicting the relationship between sex (primary exposure, green), male origin microchimerism (MOM, primary exposure, green), and mortality (primary outcome, blue) with possible ancestors of MOM (yellow), adjusted confounders (grey), and unmeasured confounders (U, red). For readability, excess arrows between confounders are omitted

In epidemiological investigations, confounding variables are often identified theoretically either a priori or guided by directed acyclic graphs, or empirically by e.g. change-in-estimate or forward/backward selection methods [19]. We averted the theoretical approaches because they require knowledge of the associations between the possible confounding variables and the exposure variable, which is not currently available for our exposure, namely MOM [20]. Likewise, empirical methods require that the decisions made about the associations are correct and do not result from sampling variability to avoid introduction of bias. Given these issues, we addressed confounding by incorporating available knowledge about factors influencing mortality, rather than MOM, as illustrated in a directed acyclic graph depicting the anticipated relationship between sex, MOM, and mortality (Fig. 1). We estimated the prognostic score [21, 22] as a single summary measure used to weigh the data and secure prognostic balance across exposure levels. Specifically, we fitted a Cox Regression model, setting death as the dependent variable, baseline predictors of death (smoking, alcohol intake, body mass index (kg/m^2^), educational attainment (a fundamental indicator of socioeconomic position shaping health related behavior across life [23]), parity, physical activity (hours/week), and dietary habits) as independent variables, and age as the underlying time scale. This model was derived using only data of women who tested negative for MOM (the unexposed). To correct for potential bias due to small sample cell sizes, we used Firth’s Penalized Likelihood (coxphf package for the R). The model coefficients for all seven predictors were retrieved, thereby allowing the prognostic score to be calculated for each woman regardless of her exposure status but for complete cases only. This prognostic score was assigned to each woman as a summary variable, informing on her risk of all-cause mortality that would have been expected, had she been MOM negative. Subsequently, we performed optimal full matching on the prognostic score between women who tested negative and positive for MOM to assign a weight to balance the two exposure groups [21, 24]. Finally, the weights were included in the Cox regression models using the survival package to obtain adjusted estimates of the association between MOM exposure and mortality. We made the R code openly available at the Open Science Framework [https://osf.io/q4ypr/?view_only=cbb0b51b548141cfaf49e6e32227c582], DOI 10.17605/OSF.IO/Q4YPR.

Characteristics of the study population are presented for women who tested positive and negative for MOM, and for men. Based on Cox regression models, we generated prognostic score weighted survival curves for women according to MOM status. Time-at-risk was counted from the time of the interview, i.e. enrolment in the cohort until death, migration, or end of follow-up, whichever occurred first. MOM was analyzed as a binary variable (positive versus negative) and estimates of association with mortality are presented as hazard ratios (HRs) with 95% confidence intervals (95% CIs). Next, we fitted Cox regression models to estimate the total effect of sex on survival according to attained age for women who tested positive and negative for MOM, and for men. These results are presented graphically as unweighted survival curves. The absolute measures of years lost in survival were calculated using the restricted mean survival time (RMST) approach [25] for women who were tested positive versus negative for MOM. RMST represents the area under the survival curve for each group and reflects the expected survival time. While including all ages would equate RMST to life expectancy at birth, in this analysis, it is limited to the years lost within the age ranges under study.

In sub-analyses, we first re-ran the association between MOM and mortality using traditional confounder adjustment rather than weighing for the prognostic score. We adjusted for the same variables used to calculate the prognostic score for comparability. Second, we calculated the E-value [26] as the minimum strength of association a group of uncontrolled confounders would need to have to explain away the associations observed between MOM and mortality. Third, mortality was evaluated according to the concentration of MOM in groups of 0, > 0 to < 2, ≥2 to < 4, or ≥ 4 MOM cells per 10⁶ cells tested. Fourth, we assessed the joint exposure to MOM and hormonal exposure at baseline on survival as previously described by our group [16] by constructing a variable with the following four levels: (1) women tested MOM positive who reported hormone exposure (nulliparity or ever use of hormonal replacement therapy or never use of oral contraceptives), (2) women tested MOM positive who reported no hormone exposure (parous and never use of hormone replacement therapy and ever use of oral contraceptives), (3) women tested MOM negative who reported hormone exposure, and (4) women tested MOM negative who reported no hormone exposure. This joint effect analysis was performed unadjusted and adjusted for smoking, alcohol intake, body mass index, educational attainment, physical activity, and dietary habits (the variables used to calculate the prognostic score, but without parity, which was included in the estimation of hormonal exposure). We assessed statistical interaction by incorporating a product term between MOM and hormonal exposure alongside their respective main effects. From the HR estimates, we calculated the relative excess risk due to interaction (RERI) with 95% CI [27]. Fifth and finally, we conducted unweighted cause-specific mortality analyses, treating death from other causes as a censoring event to appropriately account for competing risks. Results are reported as HRs, representing the relative event rates for the following comparisons: women tested MOM positive versus MOM negative, women tested MOM positive versus men, and women tested MOM negative versus men. Causes of death were categorized according to major chapters in the International Classification of Disease (version 10) system as follows: cancer (C), cardiovascular disease (I), respiratory diseases (J), neurological diseases (F, G), and other observed causes (A, B, D, E, K, M, N, R, V, W, X).

We evaluated the proportional hazards assumption using the cox.zph function in the survival package [28] correlating scaled Schoenfeld residuals for each covariate with time. Data management and statistical analysis were conducted using the R version 4.2.2 for Windows [29].

The research protocol was approved by the Danish Data Protection Agency (journal number 2011-41-6911) and by the Danish National Committee on Health Research Ethics (journal number H16021411) following national legislation.

## Results

Study population characteristics are shown for women by MOM status, and for men in Table 1. Women and men were of similar age and had similar body mass indexes and dietary habits. While men had higher educational attainment than women (10% vs. 18% with no education, and 36% vs. 11% with long education), women reported lower levels of alcohol intake (45% vs. 22% drinking 0–1 drinks/week and 14% vs. 29% drinking 7 + drinks/week), higher physical activity levels (20% vs. 33% with 0–9 h of weekly activity and 34% vs. 25% with 20 + weekly hours of activity), and they were more frequently never smokers (43% vs. 26%) compared to men. Of the 766 women, 71% tested positive for MOM. Women with detected MOM were slightly less likely than those without MOM to have ever used oral contraceptives (56% vs. 59%) and hormone replacement therapy (29% vs. 39% current users, and 54% vs. 49% never users). Other characteristics were similar between the two groups.

**Fig. 2:**
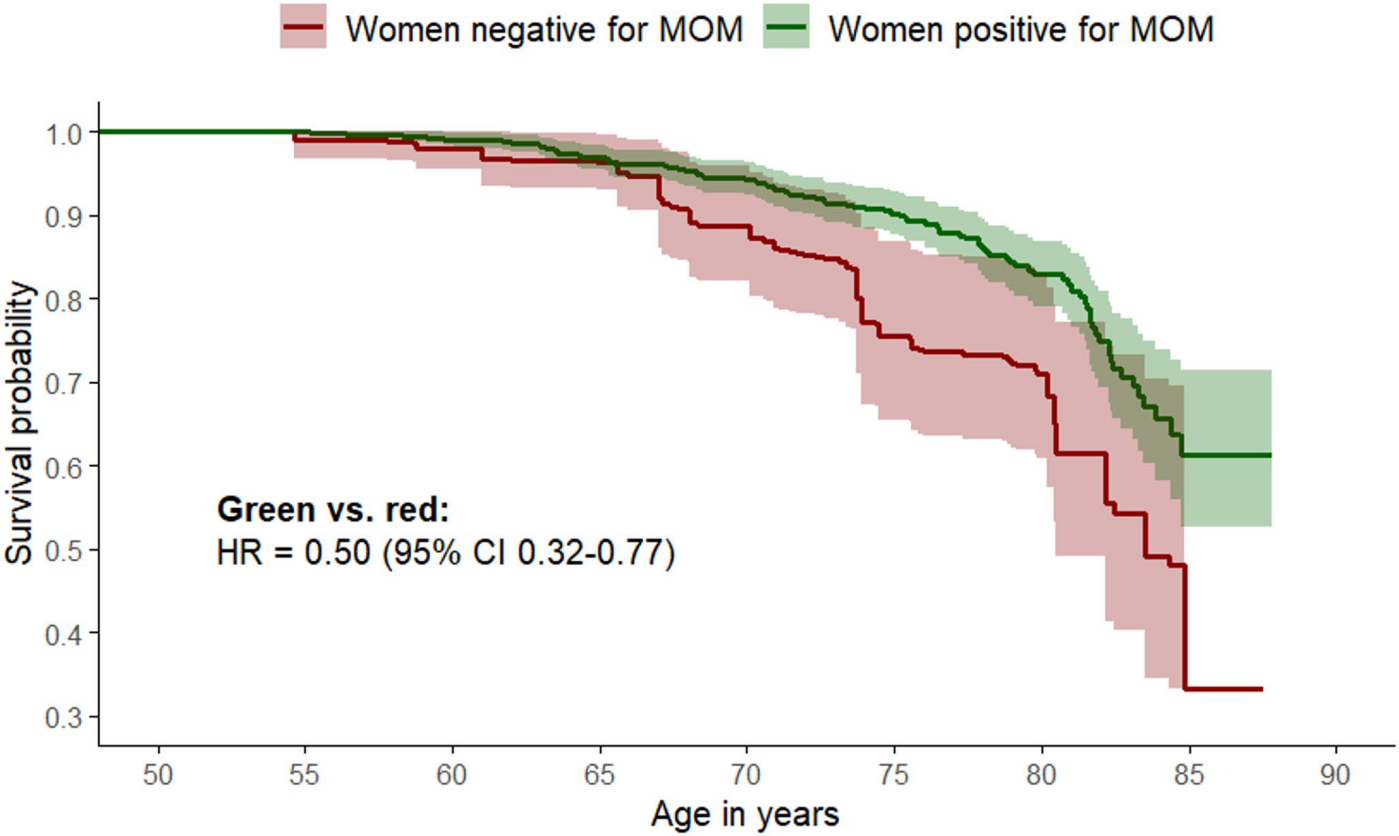
Weighted survival among women testing positive (green, *N* = 544) and negative (red, *N* = 222) for male origin microchimerism (MOM). The hazard ratio (HR) with 95% confidence interval (CI) is from a prognostic score weighted Cox regression model with age as the underlying time. The difference in restricted mean survival time (RMST difference) represents the absolute difference in years in expected survival time within the age range under study

**Fig. 3:**
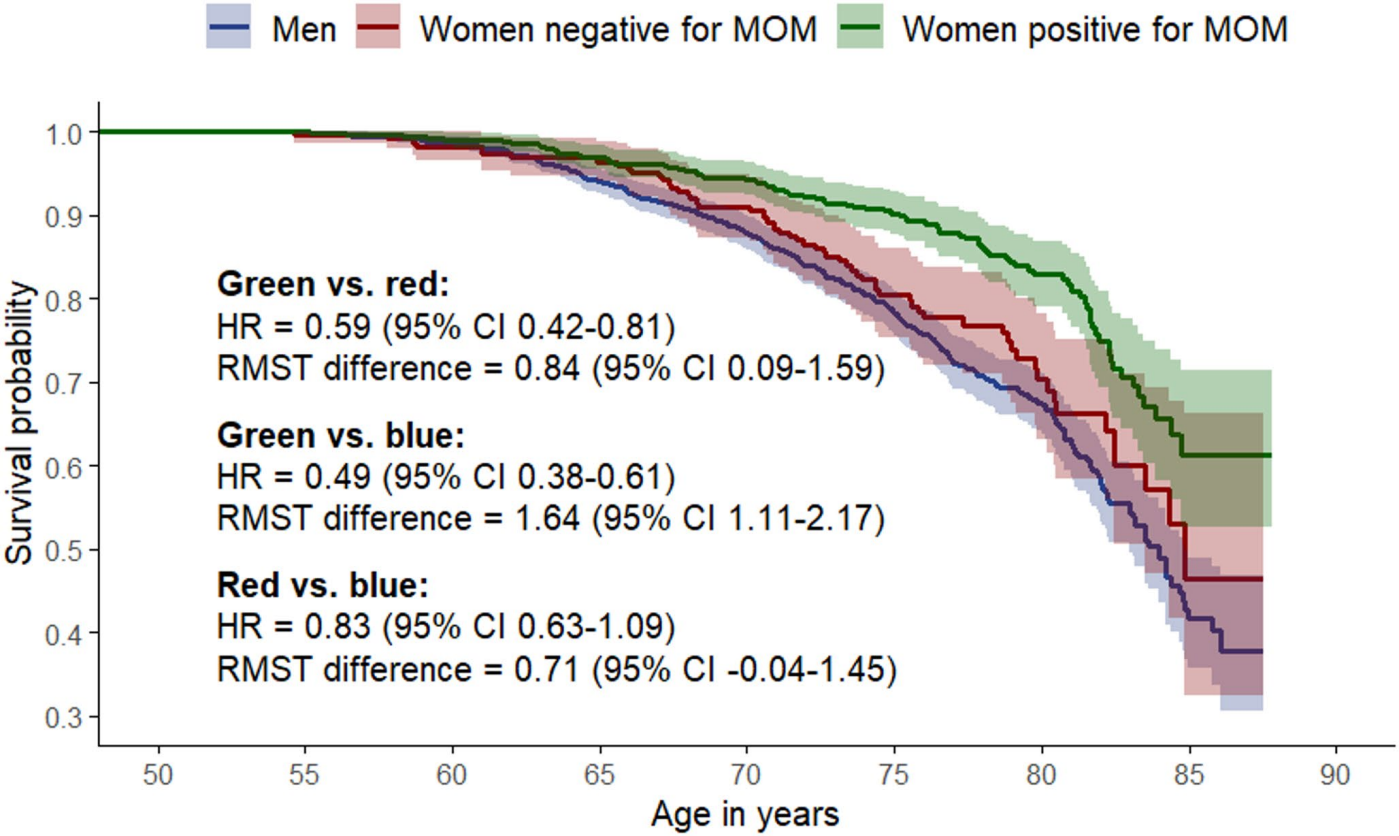
Unweighted survival among women testing positive (green, *N* = 544) and negative (red, *N* = 222) for male origin microchimerism (MOM), and among men (blue, *N* = 1,000). The hazard ratios (HRs) with 95% confidence intervals (CIs) are from Cox regression models with age as the underlying time. The difference in restricted mean survival time (RMST difference) represents the absolute difference in years in expected survival time within the age range under study

Women who tested positive compared to negative for MOM had an unweighted 41% lower mortality risk (HR = 0.59 [95% CI 0.42–0.81] which translates into an average lifespan extension of 0.84 years (95% CI 0.09–1.59, Fig. 3). After weighing for the prognostic score (calculated from smoking, alcohol intake, body mass index, educational attainment, parity, physical activity, and dietary habits), the women who tested positive for MOM had a 50% lower mortality risk compared with those who tested negative (HR = 0.50 [95% CI 0.32–0.77], Fig. 2). Traditional adjustment for potential confounders, rather than weighing for the prognostic scores, yielded a HR of 0.58 (95% CI 0.42–0.80) among MOM positive compared to MOM negative women (data not shown). During the follow-up period, 154 deaths occurred among the 766 women in the cohort, corresponding to 20%. With this relatively frequent outcome, the observed HR of 0.50 can be explained away by a group of uncontrolled confounders if they are associated with both MOM and mortality by a risk ratio of at least 2.61-fold each, beyond the effects of the measured confounders. The CI could be moved to include the null by uncontrolled confounding associated with both MOM and mortality by a risk ratio of 1.69-fold each, under the same conditions.

The estimated total effect of sex on absolute survival is shown in Fig. 3. The results for women are presented according to MOM status. Women who tested positive for MOM had a 51% lower mortality risk (HR = 0.49 [95% CI 0.38–0.61]) than men, corresponding to an average extra 1.6 years (95% CI 1.11–2.17) of life. For women who tested negative for MOM, the mortality risk was suggestively reduced by 17%, compared to that of men (HR = 0.83 [95% CI 0.63–1.09]).

Table 2 displays the association between levels of detected MOM cells per 10⁶ cells tested and all-cause mortality. Mortality was reduced among women with MOM presence at any level compared with MOM negative women. No clear differences were observed between different detected concentrations of MOM. Weighing for the prognostic score led to stronger associations with further decreased mortality of approximately 10%-points in all groups with detected MOM > 0 cells per 10⁶ cells tested.

Table 3 shows the unadjusted and adjusted associations of joint exposure to MOM and hormones with risk of death. The unadjusted results suggested that women who tested MOM positive but were not exposed to hormones had the lowest rate of death (HR = 0.36 [0.17–0.76]) compared to the reference group of women who tested MOM negative, and who were not exposed to hormones. Among women exposed to hormones, MOM status appeared to have little or no impact on mortality (MOM negative: HR = 0.78 [0.28–2.19], MOM positive: HR = 0.68 [95% CI 0.29–1.58]). The RERI suggested that the combined effect of MOM and hormones on risk of death was greater than the sum of their individual effects. After adjustment, the rate of death remained lowest among women who tested positive for MOM (not exposed to hormones HR = 0.47 [95% CI 0.21–1.05], exposed to hormones HR = 0.64 [95% CI 0.26–1.57]) whereas women who tested MOM negative, and who were exposed to hormones had a HR of 1.28 [95% CI 0.42–3.91]. The adjusted RERI consequently changed to -0.11.

Table 4 shows that cancer was the most frequent cause of death among both women (regardless of MOM status) and men followed by cardiovascular disease. Cancer-related death rates were lower among MOM positive women compared to both MOM negative women (HR = 0.54 [95% CI 0.35–0.84]) and men (HR = 0.61 [95% CI 0.43–0.85]), while cancer death rates among MOM negative women did not differ clearly from those observed in men (HR = 1.14 [95% CI 0.77–1.67]). For cardiovascular disease, women, regardless of MOM status, had lower event rates than men (MOM positive HR = 0.43 [95% CI 0.27–0.69]; MOM negative HR = 0.32 [95% CI 0.14–0.73]). Respiratory disease mortality was lower among MOM positive women than MOM negative women, while no appreciable difference was found between women and men. For neurological diseases, there was no convincing evidence of association with sex or MOM status.

We observed no violations of the proportional hazards assumption (data not shown).

## Discussion

In the present update and extension of an initial report [8], we examine the total effect of sex on survival while stratifying women by MOM status based on the hypothesis that it modifies women’s survival. In most societies, including Denmark, men have higher mortality rates than women [30]. Here, we show that the overall survival benefit of women is confined to those who test MOM positive. Focusing on MOM in women only, we find mortality to be 50% lower among women who test positive versus negative for presence of MOM in their circulation (HR = 0.50 [95% CI 0.32–0.77]). In comparison, we found a HR of 0.42 (95% CI 0.17–1.03) in the initial report. We believe that the difference reflects low statistical precision in the initial report, rather than waning of a possible beneficial role of MOM with increasing age. Alternatively, the difference could stem from selection as unknown covariates with differential effects on MOM positive compared to negative women may exist.

Previously, women have been suggested to outlive men e.g. because they are exposed to higher levels of estrogen [31] and lower levels of testosterone [32], or because the female X chromosome contains more back-up genetic material than the male Y chromosome [33]. The current report challenges these suggestions, because if true, all women regardless of MOM-status would benefit from advantageous sex-hormone levels and genetics associated with being female. Our findings suggested that detectable MOM was associated with a reduced risk of death among women regardless of their hormone exposure. The initially observed additive interaction between MOM and hormones on the risk of death seemed to be attributable to confounding.

Results from our cause-specific mortality analyses indicate that the survival advantage observed among MOM positive women relative to both MOM negative women and men is largely attributable to lower cancer-related mortality in this group. This is consistent with research indicating that women who test positive for MOM exhibit reduced that cancer morbidity, compared with women who test negative. Previous and ongoing work by our and other groups supports reduced risks of developing breast [34], ovarian [13], uterine [35], papillary thyroid [36], brain [15] and lung cancer [37]. Further, women who test MOM-positive suggestively have reduced mortality following a diagnosis of cancer [38]. Our group previously investigated associations between MOM and incident ischemic disease in women in a previous analysis and found reduced risk of ischemic events among MOM positive, compared with negative women [16]. While we find no difference in cardiovascular mortality between MOM positive and negative women in this present study, this may be caused by the small numbers of deaths due to certain causes resulting in very large and heterogeneous groups, or by mortality patterns differing in women and men complicating direct comparison. One should be skeptical if differences apply to external causes of death because no plausible link exists with MOM, but reassuringly, we found similar risk of accidents and poisoning in MOM positive and negative women (data not shown).

By weighing women according to their prognostic scores, i.e. scores summarizing characteristics that predict risk of death, to all participants, we strived to make women who tested positive and negative for MOM comparable in terms of baseline risk for the outcome. The prognostic score weighing method is particularly useful for estimating the effect of MOM on survival in women because predictors of MOM are not identified with certainty, because MOM is a common exposure, and because predictors of death are well known. We estimate the change in survival if women who tested MOM positive instead tested MOM negative. This counterfactual approach does, however, not generate causal estimates if its assumption of no hidden bias, defined as all relevant confounders included, is violated [21]. Unfortunately, we have no formal way of testing this assumption and it is likely that other confounders besides smoking, alcohol intake, body mass index, educational attainment, parity, and physical activity exist. We are not aware of any single potential confounder—or group of confounders (denoted as U in Fig. 1)—capable of inducing a 2.61-fold change in both MOM presence and mortality risk, as implied by the calculated E-value. Although residual confounding may persist, we consider it unlikely to be strong enough to fully account for the observed association. If causally linked with reduced mortality, MOM may act through increased immune surveillance as observed in allogeneic stem cell transplantation [39] and/or through tissue repair as observed in wound healing [40] and cardiac regeneration [41]. If the association on the other hand reflects confounding rather than causation, MOM may merely be a passive by-stander in women with certain immunological responses. However, MOM is not readily predicted by a range of reproductive, lifestyle, hospital or clinic visit history, and other variables [20].

MOM was detected using qPCR, which provided concentration estimates. We examined whether varying MOM levels were differently associated with mortality but found a similar reduced risk of death across all concentrations. Despite uncertainty caused by few events, this supports the notion that MOM is not associated with mortality in a dose-response manner consistent with previous reports from this cohort [8, 13, 16]. We distinguish between women who test positive and negative for MOM. Although the applied assay detects as little as 1 male cell per 100,000 cells tested [10], we may have missed MOM presence in some women, because it occurs at very low concentrations. Some evidence supports that all women pregnant with a male fetus test positive for Y chromosome in tissue [42], but oftentimes discrepancies exist between different compartments, so that women may test MOM positive in peripheral blood, but not in thyroid tissue, and vice versa [36]. Possible misclassification of MOM at time of enrolment into the DCH cohort, however, is likely not associated with later risk of dying, and therefore most likely would bias the association towards the null.

Our hormonal exposure measurement may be subject to misclassification. Data on nulliparity, hormone replacement therapy, and oral contraceptive use were based on broad, self-reported categories, limiting details on dosage and duration. Ever/never classifications complicate interpretation, as high-dose, long-term use likely influences hormone levels more than single or short-term, low-dose use. Nulliparity may be overreported due to unrecognized early pregnancy loss, increasing misclassification in that group. Although mortality cannot directly influence these errors, common causes may include socioeconomic position, health literacy, and medication adherence thus opening a path to differential misclassification which likely causes bias towards the null. Because educational attainment was incorporated into our prognostic score, we expect any resulting bias to be modest.

Although unlikely, we cannot exclude the possibility that our results are biased by selection of women with longer survival and greater propensity for MOM into the study. Compared with DCH cohort participants, non-participants are at double risk of premature death [43]. If MOM for some reason would be more common among participants, this would explain our findings. Among participating women, however, we did not observe different associations between MOM and survival in strata of parity (data not shown).

In conclusion, this study supports that women testing positive for MOM in peripheral blood live longer than their counterparts testing negative for MOM. Also, our results suggest that the survival benefit of women compared to men may be confined to women who test positive for MOM. To help establish causal pathways that may inform preventive strategies targeting sex and MOM-related differences in mortality, we propose future research in four key areas: (1) investigating the underlying determinants of MOM, including its temporal stability and site-specific variation in prevalence, (2) estimating the impact of sex and MOM status on mortality from specific diagnostic causes rather than broad categories, (3) exploring potential associations between MOM and chronic diseases relevant to mortality risk, and (4) examining associations between MOM and indicators of biological aging, such as epigenetic age, telomere length, and other relevant biomarkers.

**Table 1:** Characteristics of women and men in the study. For women, characteristics are presented overall and according to male origin microchimerism (MOM) status

	Women			Men
	All	MOM negative	MOM positive	All
N (%)	766	222 (29)	544 (71)	1,000
Age at baseline (years), median (inter-quartile range)	56 (53–60)	56 (53–60)	56 (53–60)	56 (53–60)
Age at exit (years), median (inter-quartile range)	77 (73–80)	76 (73–80)	77 (73–81)	75 (73–79)
Use of oral contraceptives, n (%)				
Ever	435 (57)	130 (59)	305 (56)	-
Never	326 (43)	89 (41)	237 (44)	-
Missing	5	3	2	-
Use of hormone replacement therapy, n (%)				
Current	243 (32)	87 (39)	156 (29)	-
Former	120 (16)	27 (12)	93 (17)	-
Never	403 (53)	108 (49)	295 (54)	-
Missing	0	0	0	-
Parity, n (%)				
0	71 (9)	22 (10)	49 (9)	-
1	122 (16)	32 (14)	90 (17)	-
2	320 (42)	101 (45)	219 (40)	-
3+	253 (33)	67 (30)	186 (34)	-
Missing	0	0	0	-
Age at first pregnancy (years), n (%)				
16–24	438 (58)	134 (61)	304 (57)	-
25–29	206 (27)	53 (24)	153 (29)	-
30+	53 (7)	15 (7)	38 (7)	-
Irrelevant	56 (7)	18 (8)	38 (7)	-
Missing	13	2	11	-
Abortions, n (%)				
Yes	225 (29)	69 (31)	156 (29)	-
No	541 (71)	153 (69)	388 (71)	-
Missing	0	0	0	-
Educational attainment, n (%)				
No vocational training	136 (18)	35 (16)	101 (19)	97 (10)
Short (< 3 years)	249 (33)	80 (36)	169 (31)	131 (13)
Medium (3–4 years)	293 (38)	89 (40)	204 (38)	407 (41)
Long (> 4 years)	88 (11)	18 (8)	70 (13)	362 (36)
Missing	0	0	0	3
Alcohol intake, drinks/week, n (%)				
0–1	348 (45)	91 (41)	257 (47)	221 (22)
2–4	229 (30)	73 (33)	156 (29)	316 (32)
5–6	80 (10)	26 (12)	54 (10)	171 (17)
7+	109 (14)	32 (14)	77 (14)	290 (29)
Missing	0	0	0	2
Smoking status, n (%)				
Current	259 (34)	87 (39)	172 (32)	422 (42)
Former	178 (23)	39 (18)	139 (26)	319 (32)
Never	329 (43)	96 (43)	233 (43)	258 (26)
Missing	0	0	0	1
Body mass index (kg/m^2^), n (%)				
< 25	401 (52)	115 (52)	286 (53)	495 (50)
≥ 25	365 (48)	107 (48)	258 (47)	505 (51)
Missing	0	0	1	0
Physical activity (hours/week), n (%)				
0–9	154 (20)	56 (25)	98 (18)	328 (33)
10–19	353 (46)	93 (42)	260 (48)	424 (42)
20+	259 (34)	73 (33)	186 (34)	247 (25)
Missing	0	0	0	1
Dietary habits (Mediterranean diet score), n (%)				
0–2	91 (12)	25 (11)	66 (12)	109 (11)
3–5	467 (61)	144 (65)	323 (60)	587 (59)
6–8	205 (27)	53 (24)	152 (28)	303 (30)
Missing	3	0	3	2

**Table 2:** Hazard ratios (HRs) with 95% confidence intervals (CIs) of death according to male origin microchimerism (MOM) concentration in women aged 50–65 years at baseline

		Unweighted	Weighted
MOM concentration per 10⁶ cells tested	n (n deaths)	HR (95% CI)	HR (95% CI)
0	180 (71)	1 (reference)	1 (reference)
> 0 to < 2	113 (14)	0.34 (0.19–0.60)	0.28 (0.15–0.51)
≥ 2 to < 4	119 (28)	0.64 (0.42-1.00)	0.53 (0.33–0.85)
≥ 4	200 (41)	0.58 (0.40–0.86)	0.48 (0.31–0.74)

Results are presented unweighted and weighted for the prognostic score (calculated from smoking, alcohol intake, body mass index, educational attainment, parity, physical activity, and dietary habits)

**Table 3:** Estimated joint effect of male origin microchimerism (MOM) and hormonal exposure on risk of death in women

Unadjusted						
MOM detection status	No hormonal exposure		Hormonal exposure		p-value for statistical interaction	RERI (95% CI)^a^
	n (n deaths)	HR (95% CI)	n (n deaths)	HR (95% CI)		
Negative	57 (14)	1 (reference)	22 (5)	0.78 (0.28–2.19)	0.189	0.54 (-0.34-1.42)
Positive	146 (13)	0.36 (0.17–0.76)	49 (9)	0.68 (0.29–1.58)		
Adjusted for smoking, alcohol intake, body mass index, educational attainment, physical activity, and dietary habits						
Negative	57 (14)	1 (reference)	22 (5)	1.28 (0.42–3.91)	0.950	-0.12 (-1.66-1.42)
Positive	146 (13)	0.47 (0.21–1.05)	49 (9)	0.64 (0.26–1.57)		

The hazard ratios (HRs) with 95% confidence intervals (CIs) are from Cox regression models with the joint effect of MOM and hormonal exposure included as one exposure variable with four levels and age as the underlying time. The results are presented unadjusted and adjusted for potential confounders with test for statistical interaction and calculated relative excess risk due to interaction (RERI) with 95% CI

^a^Calculated as: RERI = HR_hormone1_mom1_ - HR_hormone1_mom0_ - HR_hormone0_mom1_ + 1

**Table 4:** Distribution of cause-specific deaths and unweighted hazard ratios (HRs) with 95% confidence intervals (CIs) by male origin microchimerism (MOM) status and sex

Cause of death (ICD-10 chapters^a^)	Women (*n* = 766)	MOM positive women (*n* = 544)	MOM negative women (*n* = 222)	Men (*n* = 1000)	MOM positive women vs. MOM negative women	MOM positive women vs. men	MOM negative women vs. men
	Number of deaths (%)				HR (95% CI)		
Cancer (C)	79 (51)	46 (49)	33 (54)	125 (40)	0.54 (0.35–0.84)	0.61 (0.43–0.85)	1.14 (0.77–1.67)
Cardiovascular disease (I)	27 (18)	21 (23)	6 (10)	81 (26)	1.32 (0.53–3.28)	0.43 (0.27–0.69)	0.32 (0.14–0.73)
Respiratory disease (J)	16 (10)	7 (7)	9 (15)	20 (6)	0.30 (0.11–0.80)	0.57 (0.24–1.35)	1.91 (0.87–4.19)
Neurological disease (F, G)	9 (6)	5 (5)	4 (7)	20 (6)	0.48 (0.13–1.78)	0.41 (0.15–1.10)	0.87 (0.30–2.54)
Other (A, B, D, E, K, M, N, R, V, W, X)	23 (15)	14 (15)	9 (15)	69 (22)	0.59 (0.26–1.37)	0.33 (0.19–0.58)	0.55 (0.28–1.11)

^a^ International Classification of Disease (version 10) chapter
